# Sex and resting heart rate influence the relation between arterial stiffness and cardiac structure and function – insights from the general population

**DOI:** 10.1038/s41371-025-01000-0

**Published:** 2025-03-07

**Authors:** Anna Sista, Till Ittermann, Stefan Gross, Marcello R. P. Markus, Keeron Stone, Lee Stoner, Nele Friedrich, Marcus Dörr, Martin Bahls

**Affiliations:** 1 Department of Internal Medicine B, University Medicine Greifswald, Greifswald, Germany; 2 German Centre for Cardiovascular Research (DZHK), partner site Greifswald, Greifswald, Germany; 3 Department of Community Medicine SHIP-KEF, University Medicine Greifswald, Greifswald, Germany; 4 Cardiff School of Sport and Health Sciences, Cardiff Metropolitan University, Llandaff Campus, Western Avenue, Cardiff, CF5 2YB Wales UK; 5 National Cardiovascular Research Network (NCRN), Cardiff, Wales UK; 6 Department of Epidemiology, Gillings School of Public Health, University of North Carolina, Chapel Hill, NC USA; 7 Center for Health Promotion and Disease Prevention, University of North Carolina at Chapel Hill, Chapel Hill, NC USA; 8 Institute of Clinical Chemistry and Laboratory Medicine, University Medicine Greifswald, Greifswald, Germany

**Keywords:** Risk factors, Hypertension

## Abstract

Arterial stiffness, a risk factor for cardiovascular disease, can be measured using pulse wave velocity (PWV) and augmentation index (AIx). We studied sex-specific associations between carotid-femoral PWV (cfPWV), brachial-ankle PWV (baPWV), aortic PWV (aoPWV), aortic (aoAIx), and brachial (baAIx) AIx with echocardiographic parameters. Data of 1150 participants of the Study of Health in Pomerania (SHIP-Trend 1; 530 men; median age 53 years; inter quartile range (IQR) 44 to 64) were used. Echocardiography assessed common structural and functional cardiac parameters. PWV and AIx were measured using the Vascular Explorer. Multivariable linear regression models were applied. In men, a higher brAIx was related to a greater right ventricular diameter (RV) (β 0.037; CI 0.003 to 0.148). A one m/s higher baPWV was associated with a smaller RV (β −0.037; CI −0.168 to −0.021) and right ventricular outflow tract (RVOT; β −0.029; CI −0.141 to −0.026). In men, a higher aoAIx (β 0.028; CI 0.01 to 0.122) and brAIx (β 0.029; CI 0.017 to 0.13) were associated with a greater RVOT. In women, a one m/s higher aoPWV (β 0.025; CI 0.006 to 0.105) was associated with a larger RV and a one m/s higher baPWV (β −0.031; CI −0.124 to −0.001) was inversely related to RVOT. In women, PWV associated with right ventricular dimensions, while in men, baPWV and AIx were related to right ventricular parameters. This suggests potentially sex-specific relations between PWV and cardiac structure and function.

## Introduction

Cardiovascular diseases (CVD) remain a major cause of morbidity and mortality across Europe. Even though several risk factors for CVD have been identified, there are still various aspects, that need improvement. Thus, the importance of CVD for public health in Europe is still prevalent [1]. Arterial stiffness (AS) may help for CVD risk stratification and prediction of CVD, including heart failure [2]. AS may be measured using pulse wave velocity (PWV). Several studies have investigated the relationship between carotid-femoral PWV (cfPWV) and brachial ankle PWV (baPWV) with cardiac parameters [3, 4]. However, very few studies have information on central and peripheral PWV with regards to a possible association with cardiac measures.

There are multiple ways to measure PWV [5]. The majority of previous studies used cfPWV, measured by applanation tonometry as a regional measurement for central large elastic arteries [6]. In addition to the measurement of AS of the central elastic arteries, alternative assessments are available. For example, baPWV is an oscillometric measurement and an easy diagnostic tool [7, 8]. BaPWV does not only assess central AS but is a combination of central and peripheral elastic arterial properties. This combined measurement may be useful in detecting CVD at an early stage [5].

Echocardiography is used in clinical practice to identify structural and functional properties of the heart. A greater AS is generally associated with a higher systolic blood pressure which may lead to more afterload resulting in increased workload for the left ventricle [9]. Interestingly, when the associations between cfPWV and baPWV with left ventricular mass (LVM) and diastolic function were compared in 320 individuals from Taiwan, baPWV showed larger correlation coefficients with LVM and diastolic function than cfPWV. The authors speculated that the underlying reason for their observation was that baPWV is representative of a larger area of the arterial tree [10]. These results were confirmed in a small study (n = 41) from Canada, in which the authors concluded that baPWV may be able to identify left ventricular diastolic dysfunction [11]. Likewise, in 378 participants of the Guangzhou Biobank Cohort Study-CVD baPWV was associated with diastolic dysfunction [12]. Interestingly, the relation between PWV and cardiac features seems to be influenced by sex [13]. We aimed to further investigate the relationship between segment specific PWVs (cfPWV and baPWV) with cardiac structure and function. Specifically, our goal to determine which associations may be influenced by sex as well as resting heart rate. To answer our research questions we used a large dataset of the general population from Northeastern Germany called the Study of Health in Pomerania (SHIP).

## Patients and methods

### Study population

**Fig. 1:**
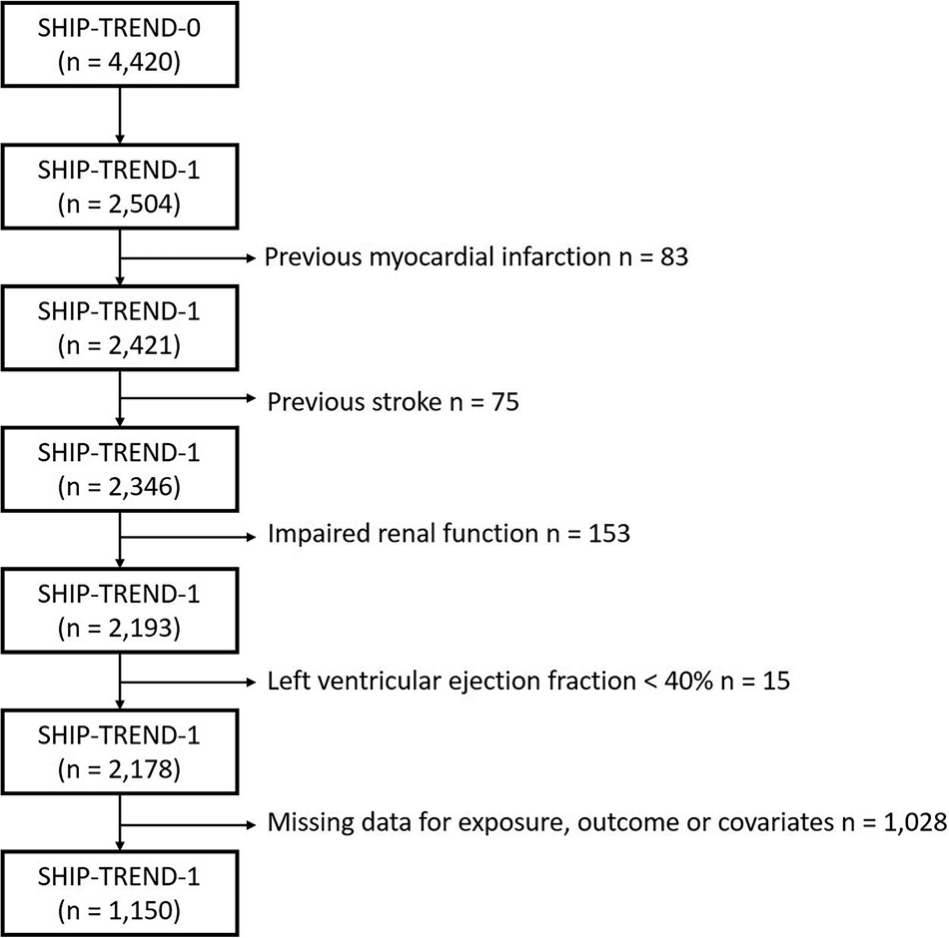
Flowchart of the study participants for The Study of Health in Pomerania Trend 1.

SHIP is a population-based study located in Northeast Germany. For SHIP-TREND 8 826 individuals were randomly selected for a health examination. These examinations took place from 2008 to 2012 and 4 420 individuals participated (response of 50.1%) [14]. The analysis in this study is based on the first follow-up of SHIP-TREND-0 called SHIP-TREND-1, in which 2 504 individuals were examined. The study design has been published elsewhere in more detail [15]. In this analysis individuals with previous myocardial infarction (n = 83), previous stroke (n = 75), impaired renal function (estimated glomerular filtration rate (eGFR) < 60 ml/min/1.73 m²) (n = 153), left ventricular ejection fraction <40% (n = 15) and missing data (n = 1 028) were excluded. The sample size was 1 150 subjects (Fig. 1). All participants gave written informed consent before taking part in the study. The study was approved by the local ethics committee and conformed to the principles of the declaration of Helsinki. SHIP data are publicly available for scientific and quality control purposes. Data usage can be applied for via https://transfer.ship-med.uni-greifswald.de/FAIRequest/.

### Pulse-wave velocity

All measurements were performed in a quiet, well ventilated, and temperature-controlled room. Study participants were placed in supine position for at least 10 min prior the measurement. Measurement was performed using the Vascular Explorer (Enverdis, Jena, Germany). Cushions were placed under the study participant’s forearm, knee and heel. All study participants were asked to avoid speaking, and encouraged to breathe calmly during the measurements. First, the circumference of the left upper arm was assessed to determine the correct size of the blood pressure cuff. A second blood pressure cuff was placed around the left ankle. The distance between the center of the blood pressure cuff on the arm to the suprasternal notch and the distance between the suprasternal notch and the middle of the blood pressure cuff on the ankle were measured using large plastic compasses. These values were entered into a software. Next a photoplethysmogram (PPG) was obtained from the left index finger and the second toe on the left foot. For the assessment of the brachial ankle index (ABI) the blood pressure cuffs on the arms and ankles were inflated to 180 and 200 mmHg, respectively. The measurement was performed automatically and based on the absence of a PPG signal. Subsequently the blood pressure cuffs were placed on the right side and the ABI measurement was performed. For the assessment of the PWV, both blood pressure cuffs were inflated to diastolic blood pressures for 15 s. During this time the pulse wave was recorded by oscillometry.

The following parameters were assessed: brachial (brSBP) and aortic systolic pressure (aoSBP); brachial (brPP) and aortic pulse pressure (aoPP); brachial (brAUG) and aortic augmentation pressure (aoAUG). Brachial and aortic augmentation indexes (AIx) were calculated by the following formula: Aix = aoAUG/aoPP. Augmentation index was normalized to a heart rate of 75 bpm.

Brachial-ankle pulse transit time (baPTT) for the calculation of baPWV was measured by device-specific algorithms detecting the real time foot-to-foot difference between brachial and ankle pulse wave. For the measurement of baPWV body height-estimated brachial-ankle travel distance were assessed. AIx and aoPWV were calculated using aoAUG, aoPP, and return time (RT) which were derived from single-point, suprasystolic brachial oscillometry (SSBO) (i.e., analysis of pulse waves recorded upon complete arterial occlusion at the brachial cuff pressure by about 35 mmHg above the systolic blood pressure). RT was measured based on the reflection method for the time difference between the beginning of the forward and reflected wave travelling acquired by decomposition of the SSBO pulse recordings. Additionally, the aoPWV values were converted into Sphygmocor-related cfPWV values using a formula provided by the manufacturer. For this reason, cfPWV is reported simultaneously with aoPWV.

### Echocardiography

Two-dimensional, M-Mode and Doppler echocardiography were performed using the Vivid-I system (GE Medical Systems, Waukesha, WI, USA) as described in detail elsewhere [16]. Measurements of LV end-diastolic and end-systolic diameter (LVDD, LVDS) and septal as well as posterior wall thickness (SWT, PWT) were performed according to the guidelines of the American Society of Echocardiography [17]. LV mass (LVM) was calculated according to the formula: LVM (g) = 0.8 × (1.04 × ((LVDD + SWT + PWT)^3^ − LVDD^3^)) + 0.6 g as described by Devereux and Reichek [18]. LVM was indexed (LVMI) for body surface area (BSA) according to Duboi (BSA = 0.20247 × height (m)^0.725^ × weight (kg)^0.425^) [18], which linearizes the relations between LVM and height.

LV wall thickness (WT), relative wall thickness (RWT), LV ejection fraction (EF) and fractional shortening (FS) were calculated following the American Society of Echocardiography [19]. Transmitral pulsed-wave Doppler was used to record early (E) and late (A) wave ventricular filling velocities. Analyses of intra-observer and inter-observer variability of echocardiographic measurements revealed high levels of agreement (absolute mean differences <8%, intra-class correlation <3%) [16].

### Medical assessments for covariates

Socio-demographic characteristics and smoking status (ever, ex-smoker and current smoker) were assessed by computer- assisted personal interviews. Height and weight were measured to calculate the body mass index (BMI = weight (kg)/height^2^ (m^2^)). Using a multifrequency Nutriguard M device (Data Input, Pöcking, Germany) and the NUTRI4 software (Data Input, Pöcking, Germany) fat free mass and fat mass were measured in participants without pacemakers. The electrodes were placed on one foot, ankle and hand. Test frequencies were 5, 50 and 100 kHz following the manufacturers instruction [20]. Waist circumference was measured to the nearest 0.1 cm using an inelastic tape midway between the lower rib margin and the iliac crest in the horizontal plane with the subject standing comfortably with weight evenly distributed on both feet. Participants with hypertension were identified by either self-reported use of antihypertensive medication (Anatomical Therapeutic Chemical Classification System [ATC]: C02, C03, C07, C08, C09) or systolic blood pressure above 140 mmHg and/or diastolic value more than 90 mmHg. Statin (ATC C10AA) medication was assessed via questionnaire and scanning of a unique drug identifier (pharmaceutical central number, Pharmazentralnummer, PZN). Laboratory covariates included levels of total cholesterol (TC), low density lipoprotein cholesterol (LDL-C), and high-density lipoprotein cholesterol (HDL-C), as well as triglycerides and glycated hemoglobin (HbA1c) that were determined photometrically (Hitachi 704, Roche, Mannheim, Germany). The criteria for type 2 diabetes were self-reported physician diagnosis, intake of hypoglycemic medication (ATC A10) or HbA1c ≥ 6.5%.

### Statistical analysis

Descriptive data are reported as absolute numbers (percentages) for categorical variables and as medians (25th and 75th percentiles) for continuous variables. To compare differences between females and males. The Wilcoxon-Mann-Whitney-Test was used for descriptive continuous variables and Chi-square for nominal variables. Parameters of AS were used as exposure variables while echocardiographic parameters were treated as outcome parameters. We used multivariable linear regression models stratified by sex adjusted for age, smoking status, mean arterial pressure, antihypertensive medication, statin use, HbA1c, height, fat mass and fat free mass. As a sensitivity analysis we also adjusted for resting heart rate. The assumption of normally distributed errors was checked by plotting residuals. A p- value < 0.05 was considered as statistically significant. STATA 18.0 (Stata Corporation, College Station, TX, USA) was used to perform statistical analyses.

## Results

### General characteristics

Our sample consisted of 1 150 participants (530 male, 46.1%) aged between 28 and 86 years (Table 1). Men had less relative fat mass compared to women. More men than women took hyperglycemic medication. There were no sex differences with regards to current smoking, but men were more often former smokers than women. The aoAIx and brAIx were lower in women compared to men. ESV and EDV were larger in men compared to women indicating a larger male heart.

**Table 1:** Description of the study population.

	Overall	Female	Male	P
Parameter	Mean (25th/75th percentile)			
Age	53 (28, 44; 63, 86)	52 (28, 44; 63, 82)	54 (28; 44; 64; 86)	0.348
Body mass index (kg/m²)	26.76 (17.59, 23.95; 29.86, 45.69)	26.05 (17.59, 23.01; 29.81, 45.69)	27.34 (17.74; 24.96; 29.92; 40.48)	0.001
HbA1c (%)	5.5 (4.0, 5.3; 5.7, 10.1)	5.5 (4.4, 5.2; 5.7, 10.1)	5.5 (4.0; 5.3; 5.7; 9.1)	0.004
Body fat (%)	33.7 (7.4, 27.0; 40.8, 59.2)	39.2 (12.6, 33.0; 45.3, 59.2)	28.2 (7.4; 22.8; 33.3; 46.9)	<0.001
Fat free mass (kg)	66.3 (40.8, 59.2; 73.0, 92.6)	60.8 (40.8, 54.8; 67.1, 59.2)	71.8 (53.1; 66.7; 77.2; 92.6)	0.549
LDL cholesterol (mmol/L)	3.3 (1.0, 2.7; 4.0, 6.8)	3.3 (1.0, 2.7; 4.0, 6.8)	3.34 (1.16, 2.7; 3.9, 6.38)	<0.001
HDL cholesterol (mmol/L)	1.50 (0.59, 1.24; 1.826.8)	1.66 (0.76, 1.41; 1.99, 6.8)	1.32 (0.59, 1.11; 1.56, 2.70)	
Smoking status				<0.001
Current (%)	18.7	18.9	18.5	
Former (%)	45.9	40.0	52.8	
Never (%)	35.4	41.1	28.7	0.313
Antihypertensive medication (%)	28.2	26.9	29.6	
Antidiabetic medication (%)	4.1	2.6	5.9	0.005
Pulse wave velocity and augmentation index				
aoPWV (m/s)	8.1 (4.0, 7.4; 9.0, 12.7)	8.4 (4.0, 7.6; 9.2, 12.3)	7.9 (4.0, 7.2; 8.6, 12.7)	<0.001
cfPWV (m/s)	9.0 (2.8, 7.8; 10.2, 15.7)	9.4 (2.8, 8.2; 10.6, 15.1)	8.5 (2.8, 7.5; 9.6, 15.7)	<0.001
baPWV (m/s)	8.6 (4.5, 7.8; 9.8, 17.2)	8.4 (4.5, 7.4; 9.5, 17.2)	9.0 (4.7, 8.0; 10.1, 16.3)	<0.001
aoAIx	17 (−13, 9; 25, 45)	21 (−3, 14; 29, 45)	11 (−13, 5; 19, 42)	<0.001
Ao75AIx	17 (−15, 10; 27, 50)	22 (−3, 15; 31, 50)	12 (−15, 5; 20, 49)	<0.001
BrAIx	−27 (−100, −51; 1, 62)	−13 (−90, −35; 13, 62)	−44 (−100, −64; −19, 55)	0.006
Echocardiography				
LVEF (%)	72.3 (41.1, 66.1, 77.7, 94.7)	72.7 (45.0, 67.4; 78.7; 93.7)	71.2 (41.1, 64.8, 77.2, 94.69)	<0.001
EDV (ml)	111 (44, 94; 133, 283)	100 (44, 86; 16, 207)	127 (45, 109; 148, 283)	<0.001
ESV (ml)	30 (6, 23; 40, 141)	27 (6, 21; 35, 97)	36 (6, 28; 46, 141)	<0.001
PWD (mm)	0.99 (0.59, 0.88; 1.10, 1.69)	0.93 (0.61, 0.83; 1.02, 1.69)	1.07 (0.59, 0.95; 1.16, 1.60)	0.004
Map (mmHg)	107 (79, 99; 115, 157)	103 (79, 96; 111, 157)	111 (82, 104; 118, 155)	<0.001
HR (bpm)	65 (36, 59; 71, 99)	66 (43, 60; 72, 98)	64 (36, 58; 70, 99)	<0.001

Continuous parameters are provided as median (minimum, 25th percentile, 75th percentile, maximum) and nominal variables are given as percentages. Differences between male and female study participants are calculated using the Wilcoxon-Mann-Whitney-Test was for continuous and Chi-square for nominal variables.

(*HbA1c* glycated hemoglobin, *LDL* low-density lipoprotein cholesterol, *HDL* high-density lipoprotein cholesterol, *aoPWV* aortic pulse wave velocity, *cfPWV* carotidal femoral pulse wave velocity, *baPWV* brachio-ankle pulse wave velocity, *aoAIx* aortic augmentation index, *ao75AIx* aortic augmentation index at 75 bpm heart rate, *brAIx* brachial augmentation index, *LVEF* Left ventricular ejection fraction, *EDV* end-diastolic volume, *ESV* end-systolic volume, *PWD* diastolic posterior wall thickness, *map* mean arterial pressure, *HR* heart rate).

### Association of AS with echocardiographic parameters

#### Association of aoAIx with cardiac parameters without adjustment for heart rate

AoAIx was positively associated with structural echocardiographic parameters including LVD, LVS as well as with ESV and inversely associated with RWT independent of sex (Tables 2 and 3). Only in women a greater aoAIx was related to a higher MV E-wave and a lower MPAP. Conversely, aoAIx was positively related EDV, SV and RVOT in men but not in women.

**Table 2:** Relationship between AIx and echocardiographic parameters in women.

	aoAIx not HR b (95 CI)		HR b (95 CI)		brAIx not HR b (95 CI)		HR b (95 CI)	
		p		p		p		p
LV structure								
LVD (cm)	**0.026 (0.003; 0.108)**	**0.04**	−0.033 (−0.044; 0.085)	0.54	**0.026 (0.003; 0.107)**	**0.04**	0.033 (−0.045; 0.084)	0.55
IVSd (cm)	0.009 (−0.029; 0.006)	0.20	−**0.011 (**−**0.045;** −**0.002)**	**0.03**	−0.009 (−0.029; −0.029)	0.21	−**0.011 (**−**0.045;** −**0.001)**	**0.04**
IVSs (cm)	0.013 (−0.034; 0.016)	0.48	−**0.016 (**−**0.062;** −**0.00)**	**<0.05**	0.013 (−0.034; 0.015)	0.46	−**0.016 (**−**0.063**−**0.001)**	**0.04**
LVS (cm)	**0.026 (0.012;0.115)**	**0.02**	0.033 **(**−0.002**;**0.125**)**	0.06	**0.026 (0.013;0.115)**	**0.01**	0.032 **(**−0.002**;**0.126**)**	0.06
WT (cm)	0.009 (−0.026; 0.008)	0.31	−**0.011 (**−**0.043;** −**0.00)**	**<0.05**	0.009 (−0.026; 0.008)	0.30	−**0.011 (**−**0.043;** −**0.001)**	**0.04**
RWT (cm)	−**0.003 (**−**0.01;** −**0)**	**0.04**	0.003 (−0.012; 0)	0.05	−**0.003 (**−**0.01;** −**0.00)**	**0.03**	−0.003 (−0.012; −0.00)	0.05
LV systolic function								
ESV (ml)	**0.669 (0.129; 2.758)**	**0.03**	0.834 **(**−0.136**;** 3.138**)**	0.07	0.667 **(**0.144**;** 2.762**)**	**0.03**	0.83 (−0.111; 3.152)	0.07
LV diastolic function								
MV E-wave (cm/s)	**0.01 (0.002; 0.039)**	**0.03**	0.012 (−0.018; 0.029)	0.63	**0.01 (0.002; 0.039)**	**0.03**	0.012 (−0.018; 0.029)	0.65
RV function								
MPAP (mmHg)	−**0.316 (**−**1.649;** −**0.409)**	**<0.01**	0.392 (−1.271; 0.268)	0.20	−**0.314 (**−**1.647;** −**0.412)**	**<0.01**	0.39 (−1.273; 0.261)	0.20

Multivariable linear regression models stratified by sex adjusted for age, smoking status, mean arterial pressure, antihypertensive medication, statin use, glycated hemoglobin, height, fat mass and fat free mass were used to relate aortal and brachial Aix with echocardiographic parameters. As a sensitivity analysis we also adjusted for resting heart rate.

Left ventricle (LV) structural: *LVM* left ventricular mass, *LVD* LV diastolic diameter, *IVSD* diastolic interventricular septum thickness, *IVSs* systolic interventricular septum thickness, *LVS* LV systolic diameter, *WT* wall thickness, *RWT* relative wall thickness, LV functional diastole: *MV E-wave* mitral valve E-wave, *MV A-wave* mitral valve A-wave, LV functional systole: *EDV* end-diastolic volume, *SV* stroke volume, *EF* ejection fraction, FS fractional shortening, *LVEF* LV ejection fraction, Right ventricle (RV) structural: *RV* Right ventricle, RV functional: *MPAP* mean pulmonary arterial pressure, *HR* heart rate.

**Table 3:** Relationship between AIx and echocardiographic parameters in men.

	aoAIx not HR b (95 CI)		HR b (95 CI)		brAIx not HR b (95 CI)		HR b (95 CI)	
		p		p		p		p
LV structure								
LVM (g)	2.678 (−0.349; 10.172)	0.07	3.335 (−5.961; 7.141)	0.86	**2.714 (0.485; 11.149)**	**0.03**	3.498 (−4.901; 8.84)	0.57
LVD (cm)	**0.03 (0.046; 0.163)**	**<0.01**	0.037 (−0.018; 0.129)	0.14	**0.03 (0.047; 0.167)**	**<0.01**	0.039 (0.023; 0.131)	0.17
LVMI(g/m^2^)	0.578 (0.045; 2.314)	0.06	0.719 **(**−1.209**;** 1.616**)**	0.77	**0.586 (0.202; 2.503)**	**0.02**	0.754 (−1.031; 1.933)	0.55
LVS (cm)	**0.032 (0.019; 0.143)**	**0.01**	**0.04 (0.009; 0.165)**	**0.03**	**0.032 (0.019; 0.145)**	**0.01**	**0.042 (0.009; 0.172)**	**0.03**
AO (mm)	0.021 (−0.029; 0.055)	0.54	**0.027 (0.001; 0.106)**	**<0.05**	0.022 (−0.034; 0.051)	0.69	0.028 **(**−0.005**;** 0.105**)**	0.07
RWT	−**0.002 (**−**0.011;** −**0.001**	**0.02**	−**0.003 (**−**0.012;** −**0.00)**	**0.04**	−**0.002 (**−**0.011;** −**0.001)**	**0.02**	0.003 (−0.012; 0.00)	0.06
LV systolic function								
EDV (ml)	**1.774 (2.623; 9.595)**	**0.01**	2.211 (−0.966; 7.72)	0.13	**1.804 (2.694; 9.784)**	**<0.01**	2.323 (−1.277; 7.85)	0.16
SV (ml)	**1.422 (0.905; 6.494)**	**0.01**	1.765 (−2.8; 4.136)	0.71	**1.446 (0.985; 6.668)**	**0.01**	1.855 (−3.133; 4.156)	0.78
ESV (ml)	**1.016 (0.413; 4.407)**	**0.02**	**1.271 (0.211; 5.207)**	**0.03**	**1.034 (0.382; 4.444)**	**0.02**	**1.336 (0.151; 5.400)**	**0.04**
LV diastolic function								
MV A-wave (cm/s)	0.008 (−0.031; 0.002)	0.09	0.01 (−0.005; 0.036)	0.15	**0.009 (**−**0.034; 0.000)**	**<0.05**	0.011 (−0.008; 0.036)	0.21
RV structure								
RV (cm)	0.036 (−0.002**;** 0.14**)**	0.06	0.045 (−0.04; 0.138)	0.28	**0.037 (0.003; 0.148)**	**0.04**	0.048 (−0.032; 0.155)	0.20
RV function								
RVOT (cm)	**0.028 (0.01; 0.122)**	**0.02**	0.035 (−0.066; 0.072)	0.93	**0.029 (0.017; 0.13)**	**0.01**	0.037 (−0.0630.083)	0.79

Multivariable linear regression models stratified by sex adjusted for age, smoking status, mean arterial pressure, antihypertensive medication, statin use, glycated hemoglobin, height, fat mass and fat free mass were used to relate aortal and brachial Aix with echocardiographic parameters. As a sensitivity analysis we also adjusted for resting heart rate. Left ventricle (LV) structural: *LVM* left ventricular mass, *LVD* LV diastolic diameter, *LVMI* left ventricular mass index, *LVS* LV systolic diameter, *AO* diameter aorta, *RWT* relative wall thickness, LV functional systole: *EDV* – end-diastolic volume, *ESV* end-systolic volume, *SV* stroke volume, LV functional diastole: *MV A-wave* mitral valve A-wave, Right ventricle (RV) structural: *RV* Right ventricle, RV functional: *RVOT* right ventricular outflow tract; *HR* heart rate.

#### Association of aoAIx with cardiac parameters adjusted for heart rate

After adjusting for resting heart rate, a higher aoAIx was associated with a smaller IVSD, IVSS and WT in women but not men (Tables 2 and 3). In male participants a higher aoAIx was related to a larger aortic diameter. The relationship between aoAIx and RWT, LVS as well as ESV were independent of resting heart rate in men but not in women.

#### Association of brAIx with cardiac parameters without adjustment for heart rate

In both sexes, a higher brAIx was associated with a higher LVD and ESV as well as a lower RWT without adjustment for resting heart rate. A higher brAIx was associated with a higher MV E-wave and a lower MPAP in women but not in men (Tables 2 and 3). In men only, we found a positive association between brAIx and LVM, LVMI, LVS, EDV, and SV. Furthermore, a higher brAIx was related to a larger RV and RVOT and a lower mitral valve A-wave only in male participants

#### Association of brAIx with cardiac parameters adjusted for heart rate

After adjusting for heart rate, a higher brAIx was inversely associated with IVSD, IVSS and WT in women but not in men (Tables 2 and 3). In men, brAIx was positively associated with LVS, RWT and ESV.

#### Association of aoPWV with cardiac parameters without adjustment for heart rate

aoPWV and left ventricular structural parameters or systolic functional parameters were not related in men or women (Table 4). A greater aoPWV was associated with a higher mitral e-wave and a larger RV only in women.

**Table 4:** Relationship between PWV and echocardiographic parameters.

	central PWV(aoPWV)	baPWV						
	not HR		HR		not HR		HR	
	b (95 CI)	p	b (95 CI)	p	b (95 CI)	p	b (95 CI)	p
Women								
LV function diastolic								
MV E-wave (cm/s)	**0.007 (0.004; 0.033)**	**0.01**	**0.007 (0.00; 0.029)**	**<0.05**	0.012 (−0.044; 0.002)	0.08	0.012 (−0.041; 0.006)	0.15
RV structural								
RV (cm)	**0.025 (0.006; 0.105)**	**0.03**	0.026 (−0.004; 0.098)	0.07	0.04 (−0.06; 0.095)	0.66	0.04 (−0.052; 0.104)	0.51
RVOT (cm)	0.02 (−0.029; 0.049)	0.62	0.02 (−0.033; 0.047)	0.73	−**0.031 (**−**0.124;**−**0.001)**	**<0.05**	0.031 (−0.123; 0.001)	0.05
Men								
LV function diastolic								
MV E-wave (cm/s)	0.007 (−0.013; 0.016)	0.87	0.007 (−0.017; 0.012)	0.75	−**0.009 (**−**0.039; 0.004)**	**0.02**	−0.016 (−0.034; 0.002)	0.08
RV structure								
RV (cm)	0.031 (−0.007; 0.113)	0.08	0.031 (−0.014; 0.107)	0.13	−**0.037 (**−**0.168;** −**0.021)**	**0.01**	−**0.038 (**−**0.16;** −**0.01)**	**0.03**
RVOT (cm)	0.024 (−0.06; 0.035)	0.60	0.024 (−0.074; 0.021)	0.27	−**0.029 (**−**0.141;** −**0.026)**	**<0.01**	−**0.03 (**−**0.123;** −**0.007)**	**0.03**
RV functional								
MPAP (mmHg)	0.288 (−0.577; 0.554)	0.97	0.287 (−0.415; 0.712)	0.61	**0.352 (0.004; 1.388)**	**<0.05**	0.355 (−0.24; 1.149)	0.21

Multivariable linear regression models stratified by sex adjusted for age, smoking status, mean arterial pressure, antihypertensive medication, statin use, glycated hemoglobin, height, fat mass and fat free mass were used to relate central and brachial pulse wave velocity with echocardiographic parameters. As a sensitivity analysis we also adjusted for resting heart rate.

Relationship between PWV and echocardiographic parameters (LV functional diastole: *MV E-wave* mitral valve E-wave, *RV* right ventricle, *RVOT* right ventricle outflow tract, *HR* heart rate.

#### Association of aoPWV with cardiac parameters adjusted for heart rate

A greater aoPWV was associated with a higher MV e-wave and a larger RV only in women (Table 4).

#### Association of baPWV with cardiac parameters without adjustment for heart rate

A higher baPWV was associated with a smaller RVOT in men and women. There was a positive association between baPWV and diastolic function only in men. A greater baPWV was associated with a lower mitral valve e-wave, a smaller RV and a higher mean MPAP (Table 4).

#### Association of baPWV with cardiac parameters adjusted for heart rate

A greater baPWV was related with a smaller RV and a smaller RVOT in men but not women (Table 4).

## Discussion

We explored the association between markers of AS assessed by oscillometry and echocardiographic measurements. Our findings indicate that aoAIx is related to structural as well as functional echocardiographic measurements in men and women. In women, aoAIx was associated with diastolic function. In men, aoAIx was positively related to a larger diameter of the aorta. Importantly, the association between PWV and cardiac markers was partly influenced by resting heart rate.

We observed differences in the association of AS with echocardiographic markers between men and women. For example, we found a positive relation between brAIx as well as aoPWV and diastolic function in women. In men, brAIx was associated with the size of the RV. This discrepancy might be attributed to the different distribution of aoAIx and brAIx being much higher in women compared to men (Table 1). In line with this, previous reports already indicated that the distribution of pulse wave parameters is different between men and women [21]. Some earlier publications suggested only slight sex-related differences on various CV biomarkers [22], others have described differences due to genetic and hormonal factors. For example, there was an association between the risk for hypertension and sex-specific genetic polymorphisms of different adrenergic receptors [23]. In determining risk factors for CVD, differentiating between men and women seems to play an important role in order to understand the diagnostical value of PWVs.

Our results highlight important differences between central and peripheral AS. For example, cfPWV is representative of the elasticity of large conduit elastic arteries and is usually measured with applanation tonometry. BaPWV, on the other hand, is measured using osscilometry and is a measure of a combination of central and peripheral arteries. Whereas in certain cardiovascular conditions such as arteriosclerosis obliterans central PWVs seem to be a better diagnostic choice due to the danger of misinterpreting peripheral PWV [24]. Peripheral PWV has not only the benefit of being a noninvasive measurement, which could entail an easier clinical use [25], but might be used as a marker for early subclinical vascular damage [5]. Both central as well as peripheral PWV might have a value in the diagnostics of CVD but the specific role for public health remains uncertain.

Based on our results the augmentation index seems to play a more important role than just PWV alone especially with regards to functional systolic parameters. The augmentation index is a known predictor of vascular health and is associated with cardiovascular events [26]. The AIx can be used as a vascular parameter but might also be influenced by cardiac parameters [27]. For example, AIx was associated with LVMI [28]. A similar result has been reported but only in men which highlights the above-mentioned importance for differentiating between the sexes [29]. This highlights the importance of the AIx and justifies further exploration of this parameter.

Our results also suggest that adjusting for resting heart rate for some but not all pulse wave related parameters is of value. Since resting heart rate is related to our exposure (i.e. PWV) and outcome (i.e. echocardiographic parameters), we adjusted our analysis for this potential confounder. A higher resting heart rate is associated with an increased risk for cardiovascular events [30]. Most previous studies adjusted their regression models for the most known risk factors for CVD excluding resting heart rate but there are a few who specifically used resting heart rate as a confounder [31–33]. Currently, there seems to be no consensus about whether to adjust for resting heart rate in studies investigating AS. However, if measures of AS are to be used for risk prediction there needs to be clear guidelines which supports future research on the importance for resting heart rate adjusted AS.

Our results are to be interpreted in light of certain limitations. The cross-sectional design does allow to draw any causal conclusions. In addition, our study population is from rural northeastern Germany. Thus, extrapolating our findings to other ethnicities is not possible as SHIP primarily includes Caucasian individuals. Lastly, there remains potential residual confounding factors that could adversely impact the results. Yet, our analysis also has several strengths. For example, a rather large well phenotyped cohort which allows for the inclusion of important confounders. The population-based cohort would suggest the applicability of our results to the general population. Furthermore, we consistently maintained a highly standardized quality control throughout the study. Another limitation is that we did not use the established Aix75 in our analysis, since we wanted to assess the influence of adjusting for heart rate in our regression models. Since Aix75 already includes heart rate, this would have invalidated our models. In the addition, the clinical relevance of some our findings needs to explored further. For example, why we identified positive associations between brAix and RV diameter but the baPWV was inversely associated with this outcome in men or whether the positive associations between aoAIx and brAIx with RVOT are clinically relevant. The pathological mechanisms of our findings remain inconclusive given the cross-sectional nature of our analysis.

In conclusion, we show that the association between AS and cardiac structure and function deserves further research. Our analysis highlights the importance of differentiating between men and women – AS may represent something different in men compared to women. This is clinically relevant given the increasing role of gender medicine for public health. In order to fully value AS for CVD prevention, future studies need to explore when an adjustment for resting heart rate is warranted.

Our findings highlight the importance of segment specific AS as an early sign of disease with a potential to indicate future cardiac function. This could potentially facilitate and improve early detection of certain cardiovascular diseases in clinical and public health settings. In turn, this could lead to increased specificity in diagnostics and treatment. However, further research into the relationship and into the underlying mechanisms involved is necessary to actually implement the measurement of AS into clinical practice.

The simplicity and non-invasive nature of PWV and AIx would be of particular benefit if used as screening techniques to detect CVDs at an earlier stage. These techniques would not require any significant additional technical expertise and could therefore be easily added to an existing clinical workflow. They are also relatively painless, which might lead to higher compliance among patients.

Our findings also highlight the importance of sex-based differences and their possible diagnostical value.

There is currently no consensus as to whether to adjust for heart rate of not. Further research comparing different analyses with and without adjusting for heart rate might give as greater insight into its significance.

## Summary

### What is known about the topic?

- Arterial stiffness is a risk factor for cardiovascular disease.
- Arterial stiffness may be determined centrally or at the periphery.

### What this study adds?

- We report differential associations between central and peripheral arterial stiffness with a broad variety of echocardiographic parameters.
- We show that sex and resting heart rate significantly influence the relationships between arterial stiffness and echocardiographic parameters in a large population based cohort.

## Data Availability

Data of the SHIP study can be obtained from the Transferstelle für Daten- und Biomaterialienmanagement (https://transfer.ship-med.unigreifswald.de/FAIRequest/data-use-intro) upon reasonable request from qualified researchers.
